# Matchmaker.com for pandas

**DOI:** 10.1093/conphys/cox033

**Published:** 2017-06-13

**Authors:** Bridie J.M. Allan

**Affiliations:** 1 Institute of Marine Research, PO Box 1870, N-5817 Bergen, Norway

Pandas are notoriously difficult to breed in captivity. Yet, a team of conservation biologists led by Martin-Wintle have demonstrated that pairing giant pandas according to their personality (panda-nality?) traits is key to successful reproduction.

Traditional panda breeding programmes focus on pairing males and females that exhibit suitable genetic differences. However, more unorthodox methods such as administering Viagra™ and exposing pandas to ‘panda pornography’ have also been used to sexually motivate them. But, what happens then these elaborate methods fail? Martin-Wintle’s team suggested that perhaps a more holistic approach is needed. Similar to how matchmaker.com works, the team investigated the role of hormone-mediated personality traits in determining mating outcomes in an iconic endangered species, the giant panda.

Personality—defined by repeatable, consistent behavioural traits—is driven by the expression of hormones and can be an important indicator of genetic and mate quality. Like humans, some animals choose partners based on behavioural traits with certain personality types that would presumably provide more effective parental care. Pairings that are based on similar traits can increase reproductive output and produce healthier offspring. A great example of this is in birds called great tits. Pairs, where male and female both display exploratory behaviours, will produce more offspring that are healthier than pairs that do not share the same exploratory tendencies. However, of these types of studies, only species that share parental care have been investigated. Little is known about the importance of personality in species where only one parent provides offspring care.

In one of the first studies to demonstrate the importance of personality traits in reproduction success in pandas, Martin-Wintle *et al.* distilled 30 behavioural traits into four main personality types: exploratory, aggressiveness, excitability and fearfulness. Pairing male and female pandas that exhibited combinations of personality types enabled the researchers to predict which personalities worked best together and resulted in successful reproduction. Martin-Wintle’s team found specific combinations of personality traits either increased or decreased reproduction, but this depended on the trait and whether the male or female exhibited it. For example, successful pairings occurred when low-aggression females interacted with high-aggression males. Aggression and competition for mates are likely driven by testosterone, an important reproductive hormone. Competition between males creates a winner/loser dynamic between individuals. The resulting ‘loser’ may have his testosterone levels suppressed, which could impact reproduction. For example, pandas that displayed fearful behaviour had limited reproductive success, which was especially obvious if a fearful male was paired with a fearless female.

When considering the importance of personality compatibility, it is surprising this ‘match-making’ approach has not received more attention in optimising captive breeding. What then is the best way to manage future panda breeding programmes? Martin-Wintle and team suggest that combining physiology, behaviour and biology could increase the number of pandas available for reintroduction into the wild. Furthermore, this approach could ensure that reintroduced pandas display favourable personality types that may lead to ‘naturally occurring’ pairings in the wild, which is the cornerstone underpinning conservation biology.

**Figure:**
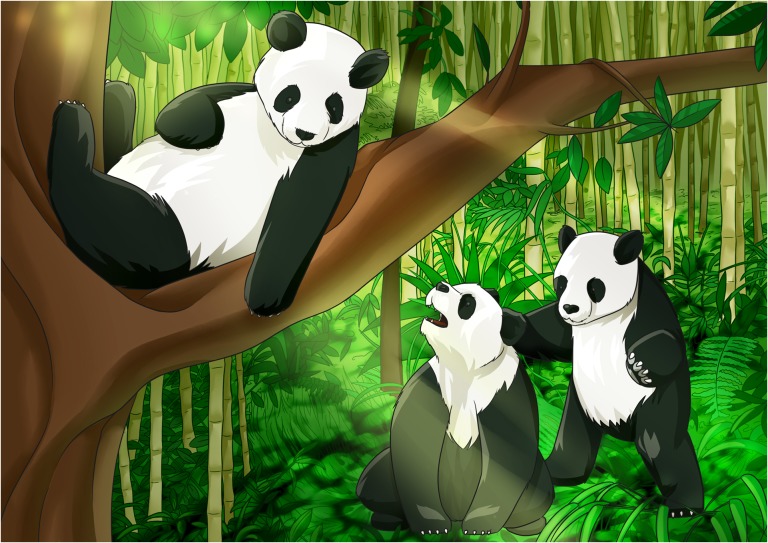


Illustration by Erin Walsh; Email: ewalsh.sci@gmail.com
